# Retraction Note: Anti-fibrotic mechanism of SPP1 knockdown in atrial fibrosis associates with inhibited mitochondrial DNA damage and TGF-β/SREBP2/PCSK9 signaling

**DOI:** 10.1038/s41420-023-01516-9

**Published:** 2023-06-30

**Authors:** Xianfeng Du, Ting Liu, Caijie Shen, Bin He, Mingjun Feng, Jing Liu, Weidong Zhuo, Guohua Fu, Binhao Wang, Yanyan Xu, Huimin Chu

**Affiliations:** 1 Arrhythmia Center, Department of Cardiology, Ningbo First Hospital, Ningbo, 315010 China; 2 Department of Nephrology, Ningbo Urology and Nephrology Hospital, Ningbo, 315000 China; 3 Ningbo University School of Medicine, Ningbo, 315211 China

**Keywords:** Cell biology, Cardiovascular diseases

Retraction to: *Cell Death Discovery* 10.1038/s41420-022-00895-9, published online 04 May 2022

The authors have retracted this article because the authors noticed a mistake in panel I of Figure 1. The incorrect raw data of the key candidate genes, including PDGFRB and CST3, was used to create the heat map. The authors no longer have confidence in the accuracy of the results presented in this article. None of the authors have responded to any correspondence from the editor about this retraction.

